# Viability and Alkaline Phosphatase Activity of Human Dental Pulp Cells after Exposure to Yellowfin Tuna Bone-Derived Hydroxyapatite In Vitro

**DOI:** 10.1155/2020/8857534

**Published:** 2020-11-23

**Authors:** Tetiana Haniastuti, Heni Susilowati, Margareta Rinastiti

**Affiliations:** ^1^Oral Biology Department, Faculty of Dentistry, Universitas Gadjah Mada, Yogyakarta, Indonesia; ^2^Conservative Dentistry Department, Faculty of Dentistry, Universitas Gadjah Mada, Yogyakarta, Indonesia

## Abstract

The bone of yellowfin tuna (*Thunnus albacares*) contains high calcium and phosphor and can be synthesized into hydroxyapatite (HA). Due to its mineral content and similarity in chemical composition with human hard tissue, HA may have potency as a pulp capping material. The aim of this in vitro study was to evaluate the viability and alkaline phosphatase (ALP) activity of dental pulp cells after exposure to HA synthesized from yellowfin tuna bone (THA). Pulp cells were isolated from human-impacted third molar. To evaluate the viability of the pulp cells, the cells were cultured and exposed to various concentrations (6.25 to 200 *μ*g/ml) of THA for 24, 48, and 72 hours. For ALP activity assay, pulp cells were cultured with odontoblastic differentiation media and exposed to THA for 7, 11, and 15 days. ALP activity was then determined using an ALP colorimetric assay kit. Results showed that the viability of the cells was more than 91% after exposure to various concentrations of THA and the cells demonstrated normal cell morphology in all observation periods. The ALP activity test revealed that groups exposed to THA for 7, 11, and 15 days showed higher ALP activity than the control groups (*p* < 0.05). It is concluded that THA had no cytotoxic effect on pulp cells; furthermore, it enhanced proliferation as well as ALP activity of the pulp cells.

## 1. Introduction

Dental pulp is a highly specialized tissue located in the center of the tooth, protected by mineralized tissue of enamel and dentin [1]. Pulp injury may occur in daily clinical practice due to cavity preparations, caries removal, or trauma. In response to the injury, pulp cells are able to regenerate and repair, forming reparative dentin [2].

Alkaline phosphatase (ALP) plays an important role in the initial formation of mineralized tissues. This enzyme stimulates pulp tissue to form dentin matrix; hence, it is essential for repair mechanisms and healing after pulpal injury. ALP activity had been identified in dental pulp cell culture [3, 4]. A study by Hayashi et al. [5] showed that growing pulp cells demonstrated low ALP activity and its activity increased as cell proliferation increased.

When pulp injury occurs, vital pulp therapy is needed to maintain pulp vitality and functions [6]. In this therapy, a capping material is applied to facilitating the healing and repair of the pulp; thus, the capping material is one of the key factors in determining the treatment outcome [7]. Various materials have previously been used as pulp capping materials. Among them, calcium hydroxide (Ca(OH)_2_) has been extensively used in clinics and has long been considered as the gold standard [8]. Many studies reported that calcium hydroxide-based materials induced hard tissue bridging; however, the presence of tunnel defects in the dentin barrier due to the porosity of the structure of reparative dentin formed by calcium hydroxide was observed [9]. It has been reported that pulp tissues became strongly irritated and a necrotic layer formed as a result of the alkaline pH of Ca(OH)_2_ [10]. An in vitro study by Alliot-Licht et al. [11] showed that human pulp fibroblast cells demonstrated alterations in cell morphology, DNA synthesis, alkaline phosphatase activity, and protein synthesis after incubation in the presence of Ca(OH)_2_. Due to these disadvantages, the development of biocompatible materials that induce a dentin-pulp complex repair is required.

Nowadays, hydroxyapatite (HA) is attracting interest as a biomaterial for use in clinical dentistry due to its similarity in crystallography and chemical composition with human hard tissue. It is one of the few materials, classified as a bioactive biomaterial, which supports bone ingrowth and osseointegration when used in orthopedic as well as dental and maxillofacial applications [12]. Due to its mineral content and similarity in chemical composition with human hard tissue, HA may have potency as a pulp capping material. An in vivo study showed that HA was a safe biomaterial for use as a dental pulp capping material and was able to induce dentinal bridge formation [13]. A previous study by Okamoto et al. [14] revealed that HA application on dental pulp developed no necrotic layers and induced less inflammation compared to Ca(OH)_2_.

Yellowfin tuna (*Thunnus albacares*) is a species of tuna found in tropical and subtropical ocean worldwide. This fish is one of the most widely consumed fish in Indonesia. The bone of the fish contains high calcium and phosphor and can be synthesized into HA [15]. This study aimed to evaluate the viability and alkaline phosphatase activity of human dental pulp cells after they were exposed to HA synthesized from yellowfin tuna bone (THA).

## 2. Materials and Methods

### 2.1. Tuna Bone-Derived Hydroxyapatite Preparation

The bone tissue of yellowfin tuna was cleaned and heated with pressure (80 kPa) for 1.5 hours and then dried with an oven at a constant temperature for 8 hours per day for 4 consecutive days. After the heat treatment, the material was ground into a fine powder. The THA powder was then sintered at 700°C for 5 hours and sieved at 200 mesh of pore size (74 *μ*m).

### 2.2. Dental Pulp Cell Culture

Normal human-impacted third molars were collected from healthy patients (18–24 years old) undergoing surgery. Teeth were removed under local anesthesia, and patients concerned gave informed consent before surgery. All protocols were approved by the Institutional Ethics Committee of Faculty of Dentistry, Universitas Gadjah Mada. Teeth surface was cleaned, and then, to reveal the pulp chamber, the cementum-enamel junction was cut around by using sterilized dental fissure burs. The pulp tissue was gently separated from the crown and root. The pulp cells were cultured in DMEM supplemented with 10% FBS, 2% penicillin and streptomycin, and 0.5% fungizone.

### 2.3. MTT Assay

Pulp cells were seeded in 96-well plates at a final density of 10^4^ cells/well in growth medium and allowed to adhere overnight. The cells were treated with different doses (200, 100, 50, 25, 12.5, 6.25, and 0 *μ*g/ml) of THA, and their viability was assessed at 24, 48, and 72 hours following treatment using MTT assay. Subsequently, the medium was removed and 200 *μ*l of MTT solution was added to the cultures. Cells were incubated at 37°C in a humidified atmosphere of 95% air and 5% CO_2_ for 4 hours. After the medium was removed, 100 *μ*l of 10% SDS was added to each well to dissolve purple crystals of formazan. The absorbance was measured using a spectrophotometer at a wavelength of 550 nm. Cell viability was expressed as a percentage of absorbance relative to the untreated cells. All experiments were conducted in triplicate.

### 2.4. Ethidium Bromide-Acridine Orange Staining

Pulp cells were seeded at a density of 10^4^ cells/well in 24-well plates with coverslips. After allowed to adhere overnight, cell cultures were exposed to 200 *μ*g/ml THA for 72 hours. The cells were then stained with ethidium bromide-acridine orange and immediately observed under a fluorescence microscope. Live cells appeared green, while dead cells were stained orange.

### 2.5. Alkaline Phosphatase Activity

Pulp cells were cultured with odontoblastic differentiation media (DMEM containing 10% fetal bovine serum, 2% penicillin/streptomycin, 0.25 *μ*g/ml fungizone, 10^−8^ M dexamethasone, 50 *μ*g/ml ascorbic acid, and 8 mM *β*-glycerophosphate) in a 6-well plate at a density of 10^5^ cells/well. After allowing to adhere overnight, the cells were cultured in the presence of various concentrations of THA (200, 100, 50, 25, 12.5, 6.25, and 0 *μ*g/ml) for 7, 11, and 15 days. At the end of the experiments, ALP activity was determined using an ALP colorimetric assay kit (Biovision, California, USA) with the procedure according to the manufacturer's instructions. The absorbance was measured using a microplate reader at a wavelength of 405 nm.

### 2.6. Statistical Analysis

The data obtained after testing the viability and ALP activity of the pulp cells were analyzed with one-way ANOVA followed by LSD with a confidence level of 95% (*p* < 0.05) considered statistically significant.

## 3. Results

**Figure 1:**
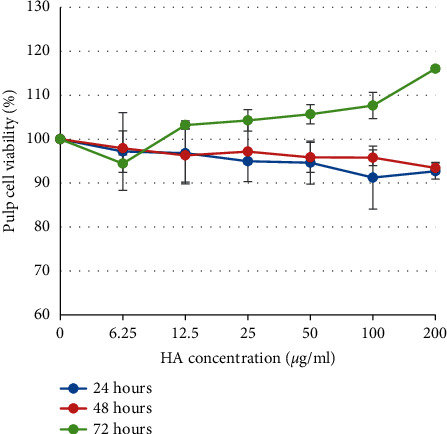
The viability of the pulp cells after treatment with different concentrations of THA (*μ*g/ml) as determined by MTT assay. Cell viability reached the highest percentage in the group treated with 200 mg/ml THA for 72 hours.

The present study showed that the viability of the cells was 91.25%–97.19% after they were exposed to THA for 24 hours and 93.44%–97.92% after they were exposed to THA for 48 hours. The viability of the cells was increased to 94.44%–116.04% after they were exposed to THA for 72 hours (Figure 1). ANOVA showed that there was no significant difference in cell viability among the groups after exposure to various concentrations of THA for 24 and 48 hours (*p* > 0.05). Those findings indicating that exposure to THA for 24 and 48 hours did not negatively influence the viability of the pulp cells. However, there was a significant difference in cell viability among the groups after exposure to various concentrations of THA for 72 hours (*p* < 0.05), indicating that exposure of THA for 72 hours affected the proliferation of the pulp cell. The MTT assay revealed a concentration-dependent increase in the pulp cell viability after exposure to THA for 72 hours. The highest concentration tested (200 *μ*g/ml) showed the highest cell viability compared to the other concentrations (*p* < 0.05) (Figure 1).

**Figure 2:**
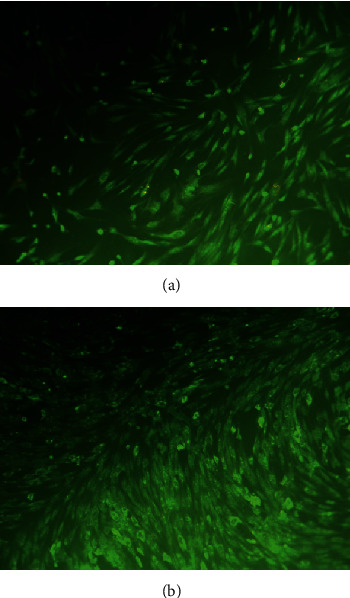
Morphology and cell density of the pulp cell culture after 72 hours stained with ethidium bromide-acridine orange: (a) untreated cells (control); (b) treated with 200 *μ*g/ml THA. The group treated with 200 mg/ml THA demonstrated higher cell density (b) than the untreated group (a).

Microscopic examination revealed that after 72 hours of culture, the pulp cells appeared as green spindle-shaped cells indicating that the cells were viable. The cells demonstrated an outstretch shape morphology (Figure 2). The cells had proliferated and showed morphological features in the form of elongated coil cells with oval-shaped nuclei, forming a linear or bundle-like formation. Neither nuclear changes nor apoptotic body formation was seen in both groups. The result showed that more cells were observed in the THA-treated group (Figure 2(b)).

**Figure 3:**
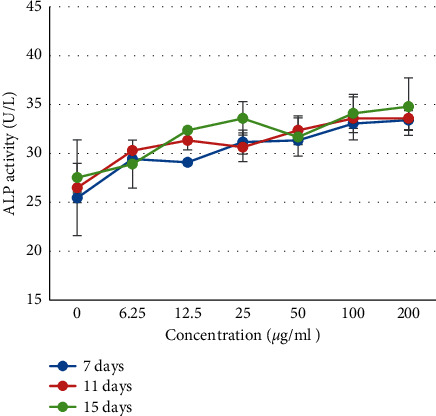
Alkaline phosphatase activity of the pulp cells after treatment with various concentrations of THA (*μ*g/ml) for 7, 11, and 15 days. The alkaline phosphatase activity was detected to increase gradually in all treatment groups.

The result of this study revealed that the ALP activity of the pulp cells increased as the concentration of THA exposure increased (Figure 3). Pulp cells treated with 100 *μ*g/ml and 200 *μ*g/ml THA showed significantly higher ALP activity than the untreated cells in all treatment periods (*p* < 0.05). Also, there was no significant difference in ALP activity between pulp cells treated with 100 *μ*g/ml and 200 *μ*g/ml THA in all treatment periods (*p* > 0.05).

## 4. Discussion

In vital pulp therapy, a pulp capping material is needed to induce reparative dentinogenesis in order to maintain pulp vitality and function [2]. This in vitro study was carried out to examine the potency of THA as a pulp capping material. The dental pulp cells used in this study were obtained from normal human-impacted third molars. A previous study had shown that pulp cells had the ability to proliferate, differentiate, and form mineralized nodules in vitro [3].

To study the effect of THA on pulp cell viability, we exposed the pulp cell culture with the various concentrations of THA. The results presented in this study showed that the viability of the pulp cell was more than 91% after exposure to various concentrations of THA in all observation periods. In addition, microscopic examination of the pulp cells after exposure to THA demonstrated normal cell morphology. Pursuant to ISO 10993-5, percentages of cell viability above 80% are considered as noncytotoxicity; thus, these findings proved that THA had no cytotoxic effect on dental pulp cells [16].

The result of the study also demonstrated that the number of viable cells treated with THA was more than the untreated control group after 72 hours of exposure. Besides, the percentage of cell viability increased with increasing concentration. Those results indicating that THA promoted pulp cell proliferation, and therefore, have proved that THA might have potency as a biocompatible and bioactive material. These might be due to the excellent biocompatibility and similarity of THA with the inorganic component of the human hard tissues [17].

The viability of the pulp cells in this study was measured with MTT assay. Concerning the chosen method, MTT assay ensures a good approximation in the study of cell viability and proliferation in cell culture since its measurement depends upon live cells [18]. The tetrazolium salt 3-(4,5-dimethylthiazol-2-yl)-2,5-diphenyltetrazolium bromide is reduced to a formazan product exclusively by mitochondrial succinate dehydrogenase enzyme in the mitochondria of viable cells; thus, the amount of formazan generated is directly proportional to the number of the viable cells [19].

ALP activity is an initial marker of odontogenic differentiation of the pulp cells. This enzyme involves in the process of mineral deposition and calcification of tissues; thus, it plays a significant role in repair mechanisms and healing after pulpal injury [20]. This study showed that ALP activity of cultured pulp cells increased with increasing THA exposure time. This finding was consistent with the previous study by Hayashi et al. [5] that isolated pulp cells showed low ALP activity, which then increased as the cells had proliferated. The results of the ALP activity test showed that groups exposed to THA showed higher alkaline phosphatase activity than the untreated group. This result suggested that THA enhanced ALP activity of pulp cells and thus might promote dentin matrix formation which is important for pulp repair after injury.

Our results proved the regenerative potential of THA in dental pulp cells with indicators of cell viability and ALP activity. These are in accordance with the results of previous research by Khojasteh et al. [21] which demonstrated that hydroxyapatite-based scaffold was able to induce proliferation and osteogenic differentiation of human dental pulp stem cells. In their study, osteogenic differentiation was indicated by an increase in the ALP activity of the pulp cells cultured on scaffold containing hydroxyapatite. Another study by Asghari et al. [22] had also shown odontogenic differentiation in human dental pulp stem cells exposed to the hydroxyapatite-coated scaffold. This was confirmed from the QRT-PCR results for genes involved in the regulation of odontoblast differentiation such as BMP2, RUNX-2, DSPP, and osteocalcin.

As a preliminary study aimed at exploring the potential of the THA as a pulp capping material, the results we have presented are evident. A limitation to our study was that biomarkers indicative of odontoblast proliferation and dentin formation such as dentin sialoprotein and collagen I should be evaluated. Besides, the complex mechanisms by which THA increases the proliferation and ALP activity of the pulp cells still need further investigation. Before use in a clinical setting, the efficacy of THA to promote reparative dentin formation on injured dental pulp should be tested in an animal model. Effects of THA on pulp inflammation and dentinal bridge formation need to be assessed in future studies. In addition, further research focusing on the characteristic of THA as a pulp capping material is also required to obtain an effective dosage form, improve physicomechanical properties, and potentiate the best biological response.

## 5. Conclusion

This study concluded that THA had no cytotoxic effect on pulp cells. Indeed, it enhances the proliferation and ALP activity of the pulp cell. Those results indicated that THA has the potency to be used as a pulp capping material. However, due to the limitation of this in vitro research, further in vivo study as well as clinical research is essential to examine the efficacy of THA to repair the injured dental pulp.

## Data Availability

The experimental data used to support the findings of this study are included within the article.
